# The additional use of methylene blue has a decatecholaminisation effect on cardiac vasoplegic syndrome after cardiac surgery

**DOI:** 10.1186/s13019-021-01579-8

**Published:** 2021-07-28

**Authors:** Walter Petermichl, Michael Gruber, Ina Schoeller, Kwahle Allouch, Bernhard M. Graf, York A. Zausig

**Affiliations:** 1 Department of Anesthesiology, University of Regensburg, Franz-Josef-Strauß-Allee 11, 93053 Regensburg, Germany; 2 Department of Anesthesiology, Aschaffenburg-Alzenau Hospital, Am Hasenkopf 1, 63739 Aschaffenburg, Germany

**Keywords:** Methylene blue, Cardiac vasoplegic syndrome, Cardiac surgery, Decatecholaminisation

## Abstract

**Background:**

Postoperative vasoplegia with minimal responsiveness to vasopressors is common after cardiac surgery. Called cardiac vasoplegic syndrome (CVS), it is caused by multiple factors. Treating CVS involves a high dose of fluids and catecholamines, however high doses of catecholamines and fluids are associated with serious side effects. There is evidence that new therapeutic strategies can lead to a reduction in norepinephrine doses and mortality in CVS. Specifically, the use of non-adrenergic vasopressors such as methylene blue (MB) can be beneficial.

**Methods:**

We retrospectively analyzed the electronic records of 8716 adult cardiac surgery patients from November 2008 to December 2016. Medication, hemodynamic and outcome parameter data were analyzed for CVS until discharge. We determined CVS according to the following parameters: a postoperative onset of ≤24 h, a reduced mean arterial pressure (MAP) of < 70 mmHg, a dose of norepinephrine ≥0.8 mg*h^− 1^ and a continuously increasing need for catecholamine, without ventricular dysfunction.

**Results:**

We identified 513 patients with CVS. Perioperative risk factors were higher in patients treated with methylene blue (MB). Before MB administration patients had a significantly higher dose of norepinephrine, and MAP increased after MB administration. Norepinephrine could be reduced after MB administration and MAP remained stable at the same level even after the reduction of norepinephrine.

**Conclusions:**

CVS patients have a severe systemic disease accompanied by significant operative stress and a high catecholamine requirement. The administration of MB in addition to standard treatment for CVS in the first 24 h was accompanied by an increase in MAP followed by a decrease in vasopressor requirement, indicating that early MB administration can be beneficial.

## Background

Postoperative hypotension is common in patients after cardiac surgery. The three major hemodynamic disorders after cardiac surgery are hypovolemia, vasoplegia and heart failure. These three disorders are responsible for episodes of hypotension that are associated with poor outcome. In particular, vasoplegia with minimal responsiveness to vasopressors such as norepinephrine, known as cardiac vasoplegic syndrome (CVS), is associated with increased mortality [1–3]. Observational studies report a CVS incidence of 5–50% in cardiac surgery with cardiopulmonary bypass (CPB) [1, 2, 4]. The routine treatment for CVS consists of administering fluids and catecholamines (e.g. norepinephrine) [2]. Catecholamines are associated with serious side effects such as increased myocardial oxygen consumption, the development of arrhythmias, or decreased renal and visceral blood flow [5]. Furthermore, the excessive administration of fluids is associated with side effects [6]. To prevent these side effects, an additional treatment to reduce catecholamine requirement is needed [7]. Observational studies report methylene blue (MB) as a therapeutic alternative or adjuvant to the classic vasoplegic syndrome therapies [1]. MB is a non-catecholaminergic agent already proven to cause a statistically significant increase in mean arterial pressure (MAP). This effect has led to no serious adverse events based on a meta-analysis of five randomized controlled trials [8]. Additionally, MB reduces catecholamine stress in critically ill patients. This effect is called decatecholaminisation and may improve survival in CVS [9, 10]. Currently, MB does not have any approved indications for CVS therapy. The purpose of this study is to determine the incidence of CPB-induced vasoplegic syndrome and to describe the practice of MB use at our cardiac surgery department. We hypothesized that MB administration in ICUs would reduce the risk of mortality in CVS patients.

## Methods

This study was approved by the University of Regensburg’s ethics committee (AZ 15101–0046). Hemodynamic records of the intensive care unit (ICU) data management system (PDMS, Metavision®, Tel Aviv, Israel) were used for this study. All adult cardiac surgery patients from November 2008 to December 2016 were screened for CVS. Cardiac vasoplegic syndrome was defined by criteria shown in Table 1. Vasopressor medication (e.g., catecholamines, hydrocortisone and vasopressin) and outcome parameters were analyzed until discharge.

**Table 1:** Vasoplegic Syndrome Definitions Criteria

Criteria	Septic CVS	Non-septic CVS
**Vasoplegic Syndrome**		
• Vasopressor requirement e.g., norepinephrine > 0.15 μg*kg^− 1^*min^− 1^	**X**	**X**
• Mean arterial pressure (MAP) ≤ 70 mmHg over 2 h or	**X**	**X**
• SVRI < 600 dyn*s*cm^− 5^*m^− 2^	**X**	**X**
• No ventricular dysfunction / CI > 2.5 L*min^− 1^	**X**	**X**
• No hypovolemia	**X**	**X**
Sepsis	**X**	**–**
Vasoplegic syndrome ≤24 h after CPB	**X**	**X**

*MAP* Mean arterial pressure, *ICU* Intensive care unit

Patients with multiple MB administration, MB administration > 24 h (h) after ICU admission, pregnant patients, patients with sepsis or patients with insufficient or missing data were excluded. Perioperative data were obtained from sources including anesthesia records (Medlinq®, Hamburg, Germany), ICU (PDMS) data, as well as medical reports and quality management (QM) data from the hospital information system (SAP®, Walldorf, Germany). The period of observation ended upon patient discharge from the hospital. Collected data were standardized and anonymized.

MB administration was set as time 0 in the MB group. Haemodynamic parameters and medications were documented at − 3, − 2, − 1 h before MB administration and at 1, 2, 3, 4, 8, 12, 24, 48 and 72 h afterwards. For the non-MB group, time point 0 was defined as 6 h after ICU admission, corresponding to the mean time of MB administration after MB-group patient admission to the ICU.

Data were analysed with SPSS (IBM Corp., Armonk. USA, version 23) using Pearson’s chi-squared test or t-tests. A *p*-value of < 0.05 was considered statistically significant. All data in the text, tables and figures are given as percentages (%), mean and standard deviation (SD) or standard error of the mean (SEM).

## Results

**Fig. 1:**
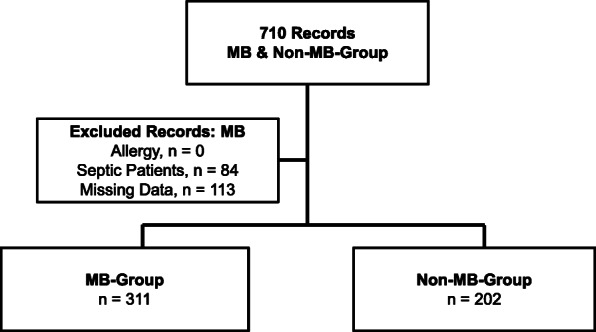
Trial Profile of the Study. Trial profile of the study showing both groups at the end: MB group and non-MB group

**Fig. 2:**
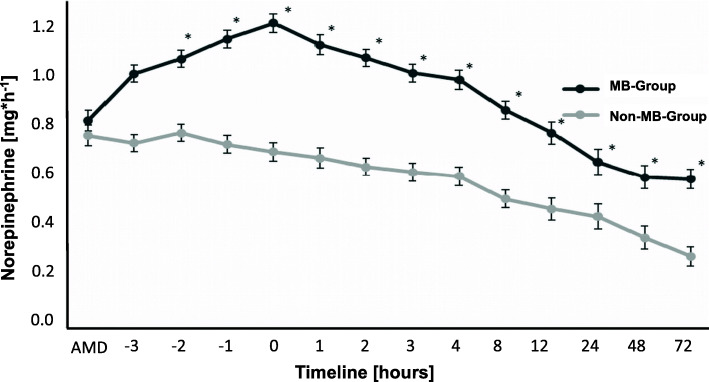
Timeline of Norepinephrine Doses in MB and Non-MB Groups. Norepinephrine dose throughout the analysis time for MB-group and non-MB-group. ADM: Admission at ICU; 0: administration of MB. All data are expressed as mean +/− SEM. *p < 0.05

**Fig. 3:**
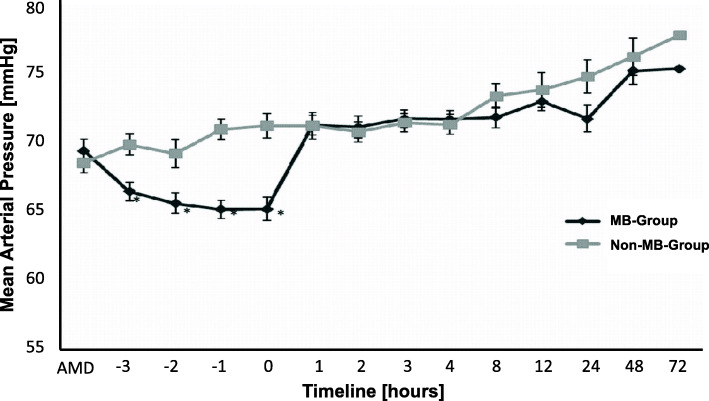
Timeline of Mean Arterial Pressure in MB and Non-MB Groups. Mean arterial blood pressure (MAP) throughout the analysis of MB and non-MB groups. ADM: admission into ICU; 0: administration of MB. All data are expressed as mean +/− SEM. *p < 0.05

From November 2008 to December 2016, our department performed 8716 cardiopulmonary bypasses (CPB). According to the definition in Table 1, a total of 710 of these CPB patients, equating 8.15% of our study population, had vasoplegic syndrome (Fig. 1). We excluded 113 patients from further analysis due to insufficient or missing data. Of the remaining 597 patients with vasoplegic syndrome, we identified 84 as septic vasoplegic syndrome patients and therefore excluded them from further analysis. Five hundred thirteen remaining patients with complete data sets fulfilled our criteria for cardiac vasoplegic syndrome. This corresponded to 5.86% of all patients after cardiac surgery. Of these 513 patients, 311 (60.23%) received MB in the first 24 h after ICU admission (MB group). The remaining 202 patients had no MB administration (non-MB group, Table 2). Demographic and intraoperative data of the MB group and the non-MB group are shown in Tables 3 and 4. Compared to the non-MB group, patients in the MB group had markedly more comorbidities and perioperative complications, e.g., kidney failure, acute myocardial infarction, emergency operation, previous cardiac surgery events, and prolonged CBP-time, among others. The MB group patients had a norepinephrine mean continuous infusion rate of 1.2 ± 0.61 mg*h^− 1^ (mean ± SD) before MB administration. In the non-MB group the continuous infusion rate of norepinephrine was 0.64 ± 0.51 mg*h^− 1^ (mean ± SD). The continuous infusion rate of norepinephrine was significantly higher in the MB group compared to the non-MB group at time 0 (*p* < 0.05; Fig. 2). Mean arterial pressure (MAP) was significantly lower in the MB group before MB administration compared to the non-MB group (*p* < 0.05; Fig. 3). Additional vasopressors such as epinephrine, vasopressin and hydrocortisone were administered significantly more in the MB group (*p* < 0.001), as shown in Table 5. Extended hemodynamic monitoring was used significantly more in the MB group compared to non-MB group. Trans-oesophageal echocardiography was performed significantly more in the MB group (160 patients, 53%) compared to the non-MB group (51 patients, 25%; *p* < 0.001). A pulmonary artery catheter (PAC) was employed 182 times (61%) in the MB group and 38 times (19%) in the non-MB group (*p* < 0.001). Pulse contour cardiac output (PICCO) was only performed in the MB group (2%). Mean central venous saturation (ScvO_2_) in the MB group (66 ± 24%) was not significantly different compared to the non-MB group (65 ± 70%). Cardiac index (CI) was not significantly different between the MB (3.3 ± 1.0 L/min/m^2^) and non-MB (3.4 ± 1.0 L/min/m^2^) (mean ± SD) groups. MB was administered 6.4 ± 5.2 (mean ± SD) h after ICU admission, with a dose of 174 ± 56 mg (mean ± SD). This dose equates to approximately 2.0 mg*kg^− 1^ body weight. In 89% of all cases MB was administered continuously by infusion pump over 51 ± 28 min (mean ± SD) and in the remaining 11% of cases MB was given as a bolus. MAP increased significantly from 65 + 0.5 mmHg (mean ± SEM) at time point − 1 to 71 + 0.5 mmHg (mean ± SEM) at time point 2 (*p* < 0.05) after MB administration. Compared to time points − 3, − 2, − 1 and 0, MAP increased significantly in the MB group at time point 1 (*p* < 0.05) (Fig. 3). No difference in the MAP levels of the MB and non-MB groups was observed beyond time point 1 to the end of the observation period. The continuous norepinephrine infusion rate decreased from time point 1 while maintaining constant hemodynamic and MAP values. At all times, the continuous norepinephrine infusion rate of the non-MB group was significantly lower than that of the MB group. The 30-day mortality was significantly higher in the MB group compared to the non-MB group (*p* < 0.001). The rates of organ failure (*p* < 0.001) and number of ventilation days were also significantly higher (*p* < 0.001) in the MB group (Table 6).

**Table 2:** Determination Criteria for Methylene Blue (MB) Group and Non-Methylene Blue (Non-MB) Group

MB Group	Non-MB Group
• Single MB administration in ICU • MB administration in ICU ≤ 24 h after arrival	• No MB administration in ICU • Norepinephrine ≥0.8 mg/h at least once in ICU ≤ 24 h after admission

*MB* Methylene blue, *ICU* Intensive care unit

**Table 3:** Demographic Data

	MB Group ***n*** = 311	Non-MB Group ***n*** = 202	***p***-value
**Age [years]**	65.7 ± 12.0	65.8 ± 11.4	n.s.
**Male**	243 [78]	163 [81]	n.s.
**BMI [kg/m**^**2**^**]**	31.7 ± 57.8	27.7 ± 6.5	n.s.
**ASA**			
**1–2**	5 [2]	8 [4]	0.001
**3**	136 [46]	122 [60]	
**4**	138 [47]	67 [33]	
**5**	12 [4]	1 [1]	
**Nicotine Abuse**	94 [32]	71 [35]	n.s.
**Coronary Heart Disease**	191 [65]	144 [71]	n.s.
**With Stent**	22 [7]	11 [5]	n.s.
**Angina Pectoris**	67 [23]	79 [39]	0.001
**Previous Myocardial Infarction**	81 [27]	35 [17]	0.009
**NYHA**			
**1**	1 [0.3]	0	0.014
**2**	15 [5]	7 [4]	
**3**	162 [56]	143 [71]	
**4**	106 [36]	47 [23]	
**Left Ventricular Ejection Fraction [%]**	47.5 ± 18.8	45.8 ± 17.	n.s.
**Pacemaker**	23 [8]	13 [6]	n.s.
**Hypertension**	203 [69]	147 [75]	n.s.
**Peripheral Arterial Disease**	47 [16]	36 [18]	n.s.
**With Stent**	25 [8]	17 [8]	n.s.
**Pulmonary Hypertension**	30 [10]	16 [8]	n.s.
**Renal Failure**			
**GFR > 89 mL/min**	35 [11]	42 [21]	0.004
**GFR 60–89**	64 [21]	58 [29]	
**GFR 30–59**	59 [19]	38 [19]	
**GFR < 30**	29 [9]	9 [5]	
**Dialysis**	1 [0.3]	0	
**Cerebrovascular Events**	20 [7]	6 [3]	n.s.
**Allergy**	54 [18]	39 [19]	n.s.

*BM* Body mass index, *ASA* American Society of Anesthesiologists, *NYHA* New York Heart Association, *GFR* Glomerular filtration rate; All data are presented as number [%] or mean +/− SD. n.s.: not significant

**Table 4:** Intraoperative Data

	MB Group ***n*** = 311	Non-MB Group ***n*** = 202	***p***-value
**Emergency Operation**	137 [44]	60 [30]	0.002
**Surgery Type**			
CABG	135 [46]	111 [55]	n.s.
Valve	54 [18]	29 [14]	
CABG+VALVE	47 [17]	19 [9]	
Aorta	5 [2]	1 [1]	
VAD	10 [3]	1 [1]	n.s.
Others	53 [18]	42 [21]	
**With CBP**	273 [88]	172 [85]	n.s.
**Previous Non-Cardiac & Cardiac Surgery**	98 [32]	32[16]	0.001
**CBP Time [min]**	119 ± 68	102 ± 62	0.006
**Cross-Clamp Time [min]**	77 ± 49	64 ± 43	0.003
**Hypothermia**	24 [8]	8 [4]	n.s.
**CPR**	51 [17]	26 [13]	n.s.
**ECMO**	20 [7]	3 [2]	0.022
**ECMO [min]**	39 ± 51	0	n.s.
**IABP**	30 [10]	7 [4]	0.035
**MAP [mmHg]**			
Pre Induction	87 ± 18	90 ± 15	n.s.
Post Induction	78 ± 13	80 ± 11	n.s.
Postoperative in OR	68 ± 15	69 ± 16	n.s.
**Heart Rate [1/min]**			
Pre Induction	79 ± 21	78 ± 17	n.s.
Post Induction	74 ± 21	71 ± 17	n.s.
Postoperative in OR	98 ± 20	96 ± 19	n.s.
**Diuresis [mL]**	812 ± 608	881 ± 627	n.s.
**Norepinephrine [mg/h]**			
After CBP	0.49 ± 0.8	0.33 ± 0.4	0.004
Before CPB	0.57 ± 0.5	0.72 ± 0.9	0.032
**Dobutamine [mg/h]**			
After CBP	4.8 ± 7.5	3.6 ± 6.3	n.s.
Before CPB	6.9 ± 9.3	8.3 ± 9.1	n.s.
**Epinephrine [mg/h]**			
After CBP	0.11 ± 0.5	0.03 ± 0.2	0.007
Before CPB	0.31 ± 0.3	0.21 ± 0.3	0.001
**Vasopressin [I.E./h]**			
After CBP	0.03 ± 0.5	0.01 ± 0.1	n.s.
Before CPB	0.31 ± 2.1	0.07 ± 0.4	n.s.
**Hydrocortisone**			
Bolus [mg]	73 ± 44	96 ± 15	n.s.
Continuous [mg/h]	13 ± 21	65 ± 60	n.s.
**Blood Products**			
Erythrocytes	2.2 ± 2.6	1.4 ± 2.1	0.001
Fresh frozen plasma	2.1 ± 3.9	1.0 ± 2.5	0.001
Thrombocytes	1.0 ± 1.4	0.7 ± 1.0	0.002
Cellsaver-blood [ml]	389 ± 742	289 ± 443	n.s.
PPSB [I.E.]	1088 ± 1867	873 ± 1547	n.s.
Minirin [μg]	9.7 ± 36.6	5.9 ± 11.3	n.s.
**Fluids [mL]**			
Crystalloids	1286 ± 959	1886 ± 1955	0.001
Colloids	449 ± 458	386 ± 445	n.s.

*CAPG* Coronary artery bypass grafting, *VAD* Ventricular assist device, *CPB* Cardiopulmonary bypass, *CPR* Cardiopulmonary resuscitation, *ECMO* Extracorporeal membrane oxygenation, *CPB* Cardiopulmonary bypass, *IABP* Intra-aortic balloon pump, *MAP* Mean arterial pressure, *OR* Operation room. All data are presented as number [%] or mean +/− SD. n.s.: not significant

**Table 5:** Additional Vasopressor Administration

	MB Group n =	Non-MB Group n =	***p***-value
**Epinephrine**	254	115	0.001
**Vasopressin**	40	6	0.001
**Hydrocortisone**	244	34	0.001

Additional vasopressor administration in MB and non-MB groups at time point 0

**Table 6:** Outcome Data

	MB Group ***n*** = 311	Non-MB Group ***n*** = 202	***p***-value
**30-Day Mortality**	57 [18]	10 [5]	0.001
**Stay**			
**ICU [d]**	26 ± 29	16 ± 16	0.001
**IMC [d]**	15 ± 18	6 ± 8	0.001
**GW [d]**	6 ± 19	4 ± 7	n.s.
**Renal Failure**	130 [42]	30 [15]	0.001
**Renal Replacement Therapy**	149 [48]	34 [17]	0.001
**Respiratory Failure**	81 [27]	6 [3]	0.001
**Liver Failure**	22 [7]	4 [2]	0.007
**First Defecation [d]**	3.6 ± 2.2	2.3 ± 1.8	0.001
**Cerebrovascular Events**	44 [15]	10 [5]	0.001
**Multi-Organ Failure**	40 [13]	8 [4]	0.001
**Sepsis**	26 [9]	9 [45]	n.s.
**TISS10**	172	163	
ADM	23.2 ± 8.7	20.1 ± 6.9	0.001
MAXIMUM	30.2 ± 6.8	24.2 ± 6.6	0.001
**SAPSII**	140	163	
ADM	24.19 ± 13.62	21.91 ± 12.13	n.s.
MAXIMUM	35.52 ± 17.85	46.18 ± 252.34	n.s.
**Ventilation Time [h]**	246 ± 900	60 ± 101	0.001
**Vasopressor-Free Days in ICU [d]**	12.3 ± 26.8	11.0 ± 12.4	n.s.

*ICU* Intensive care unit, *IMC* Intermediate care unit, *GW* General ward, *TISS* Therapeutic intervention scoring system, *SAPSII* Simplified acute physiology score. All data are presented as number [%] or mean +/− SD. n.s.: not significant

## Discussion

To the best of our knowledge, this retrospective study contains the highest number of CVS patients treated with MB of studies to date. Vasoplegic syndrome after cardiac surgery is an independent mortality factor in cardiac surgery patients [3], and CVS is caused by multiple factors [2, 11]. There are currently no standardized definition criteria for CVS [12]. For this reason, CVS incidence ranges from 5 to 50% in the literature [2, 4]. Van Vessem et al., for example, observed a vasoplegic syndrome incidence of 29% in their study [4]. Using the vasoplegia criteria shown in Table 1 and including septic patients, we identified a vasoplegic syndrome incidence rate of 8.15% (710 of 8716). Excluding sepsis as the cause of vasoplegic syndrome and excluding incomplete data sets, CVS incidence in our study population was 5.88% (513 of 8716). This incidence rate is comparable to findings from other studies investigating CVS [11, 13]. Typical risk factors (RF) for vasoplegic syndrome after CBP in these studies included CBP time, emergency operation, and previous myocardial infarction. We also identified these RF in our study (Tables 3 and 4) [2, 4, 11]. In addition to identifying possible RF, it is important to define CVS in order to facilitate early recognition and treatment. In our opinion, a combination of the most frequently used vasoplegia criteria from CVS studies offers a starting point to define CVS. These CVS criteria are: the need for increasing doses of vasopressors (> 0.15 μg*kg^− 1^*min^− 1^ norepinephrine), mean arterial pressure < 70 mmHg, a systemic vascular resistance index (SVRI) < 600 dyn*s*cm^− 5^*m^− 2^, and the exclusion of ventricular failure (CI > 2.5 L*min^− 1^) [14–17]. Patients who met all these criteria within 24 h post-CPB, whose vasoplegia was not caused by sepsis and who did not have hypovolemia, had non-septic CVS according to our extended definitions (Table 1).

The first positive hemodynamic effect of MB in cardiac surgery was reported 1996. In that study Andrade et al. reported using 1.5 mg*kg^− 1^ of MB in six patients with vasoplegic syndrome. After MB administration, SVRI increased from 868 to 1693 dyn*s*cm^− 5^*m^− 2^ [18]. It is supposed that MB’s vasoconstrictive effect is caused by two mechanisms: MB inhibits inducible NO synthase, thus reducing the vasodilating ligand, and MB binds competitively to the heme group of guanylate cyclase, thus additionally reducing the receptor binding site for vasodilation [1, 12, 19]. The result is catecholamine-independent vasoconstriction. The administration of MB can therefore reduce catecholamine requirement and catecholaminergic stress in critically ill patients. This therapeutic approach is known as decatecholaminisation [8]. But insufficient hemodynamic effects after MB administration have also been reported. MB’s variable vasoconstrictive effect may be explained by the non-standardized administration of MB; in these studies the time point of MB administration, dosage etc. differed considerably, thereby explaining the various reactions to MB [2, 5, 20, 21]. There are some side effects and complications associated with MB administration. These undesirable MB effects can be categorized into minor and severe complications. Classic minor side effects such as dizziness and nausea were not detectable in any of our patients, though these minor side effects may not have been apparent in our study as all patients were intubated and sedated during MB treatment. The administration of MB can also result in microcirculation disorders and local cutaneous necrosis [22]. Whether there is a beneficial or harmful MB effect on mesenteric perfusion in vasoplegic shock is still unclear [23]. The use of MB in glucose-6-phosphate dehydrogenase-deficient patients triggers severe hemolytic crisis in these patients and therefore must be avoided [24].The mean MB dose in our study was 2.0 mg*kg^− 1^, which corresponds to that of others studies and is described as a safe standard dose [25].In our study, a small group of twelve patients received an unintentionally higher dose of MB (4.5 mg*kg^− 1^ - 5.5 mg*kg^− 1^). Neither the standard dose group (< 2.0 mg*kg^− 1^) nor the maximum dose group (4.5 mg*kg^− 1^ - 5.5 mg*kg^− 1^) experienced any minor or serious side effects in our study.

A significant increase of 5 mmHg MAP after MB administration was detected in our study (Fig. 3), and MB administration was followed decreased vasopressor requirement (Fig. 2). These findings are comparable to those of other studies which describe the catecholamine-reducing effect of additional MB therapy in cardiac vasoplegic syndrome. Surprisingly, a reduction in mortality after MB administration in CVS was not identified in this study [13]; the mortality rate of the MB group was significantly higher compared to the non-MB group (18% vs. 5%, *p* < 0.001). These different findings are explained by our study design: the purpose of this study was to determine CVS incidence and to describe MB use in our cardiothoracic surgery department, therefore we conducted the study as a retrospective analysis. Because MB administration for severe CVS is an established treatment at our department, the two CVS groups we formed retrospectively (MB and non-MB) were not entirely comparable. When considering the noradrenaline dosage course and MAP course (Figs. 2 and 3) in the MB and non-MB groups, a lower vasopressor requirement and higher MAP in the non-MB group was detected. Typical risk factors for vasoplegic syndrome, such as comorbidities, long CBP time and emergency operation, were also significantly more frequent in the MB group (Tables 3 and 4). This indicates that patients in the MB group were at a greater risk of severe CVS compared to the non-MB group. Furthermore, we postulate that the non-MB group in our study had less vasoplegia. The incidence rates of 5–50% in the literature and the lack of a standard definition of vasoplegic syndrome confirm the thesis that there are several degrees of vasoplegia [1, 17, 26]. The MB group patients’ higher mortality rate can therefore be explained by their greater degree of illness compared to the non-MB group patients.

It is not only the severity but also the duration of vasoplegia which influences outcome. Gomes et al. demonstrated that a high mortality rate of 25% is associated with vasoplegic syndrome lasting > 36–48 h [20]. In our study with a mortality rate of 18%, MB was given early after ICU admission. After MB administration the need for norepinephrine decreased over the first hours to a level below 0.8 mg*h^− 1^ (Fig. 2). Within the first hour after MB administration, MAP increased in treated patients while vasopressor requirement decreased (Fig. 3).

Some studies have demonstrated that the use of MB in severe vasoplegic syndrome reduces the mortality rate from 44 to 21.2% [17, 27].The reduction of mortality in these studies is comparable to our study’s mortality rate of 18% in the MB group. Mehaffey et al. demonstrated that not only the administration itself but also the timing of MB administration is important in order to decrease mortality. Mehaffey’s study demonstrated that MB administration during operation reduced the incidence of postoperative renal failure and operative mortality when compared to ICU MB administration (10.4% vs. 28.6%) [13]. Other studies have also demonstrated that preoperative MB administration in patients with a high risk of vasoplegic syndrome during cardiac surgery can prevent CVS. In this study no vasoplegic syndrome in the MB-treated group was detected compared to 26% CVS in the non-MB group [25]. It is assumed that a therapeutic regime with MB as an “on-top” medication in high-risk patients is reasonable and can reduce mortality. In order to reduce CVS mortality, CVS therapy must follow the principles of therapy for sepsis: hit hard, hit early. Therefore, early administration of MB is beneficial [13, 17].

This study has some limitations. First of all, as there is no standard definition of vasoplegic syndrome, each study has different parameters for the study population. For example, Ozal et al. defined CVS mainly through surrogate parameters as MAP < 50 mmHg, cardiac index > 2.5 L*min^− 1^*m^− 2^, right atrial pressure < 5 mmHg, left atrial pressure < 10 mmHg and reduced SVR < 800 dyn*s^− 1^*cm^− 5^ throughout intravenous infusion of norepinephrine (≥ 0.5 μg*kg^− 1^*min^− 1^) [25]. In contrast, Weiner et al. defined CVS by a high dependency on the norepinephrine > 0.2 μg*kg^− 1^ min^− 1^ and vasopressin > 2 I.E.*h^− 1^ catecholamines [28]. Therefore, a comparison of the results of each study has to be done carefully. We use a combination of Ozal et al.’s and Weiner et al.’s parameters in our study: at the time of MB administration, vasoplegic patients showed a MAP > 60 mmHg, a norepinephrine dose of 1.2 +/− 0.6 mg*h^− 1^ and SVR > 800 dyn*s^− 1^*cm^− 5^. While our norepinephrine doses correlate with those defined in the other studies, MAPs and SVRs in our study were higher than in other studies [29]. This may be due to a timely therapeutic response to drops in blood pressure. A marked ramping up of norepinephrine in the first hours after admission (exaltation of 0.1 mg*h^− 1^ in at least 3 consecutive steps) is apparent in our study, which – in our view – characterizes the very unique clinical catecholamine refractory vasoplegic situation leading to MB administration. In our clinical practice, we use mg*h^− 1^ as the dosage designation. An additional dosage recalculation into the commonly used μg*kg^− 1^*min^− 1^ had no effect on the statistical statements.

Another limitation due to this study’s retrospective design is the lack of a perfect control group with the same severity of illness as the MB group. The retrospective analysis demonstrated that the non-MB group is not a true control. Tables 1 and 2 show that the MB group patients had a higher ASA classification, more emergency operations, increased norepinephrine doses before and after operation, and longer CPB and ischemia times. It is therefore not surprising that morbidity, mortality and organ failure in the MB group was higher than in the non-MB group.

## Conclusions

Cardiac vasoplegia patients suffer from a severe systemic disease accompanied by operative stress and a high requirement of catecholamines. Additional MB administration to the standard treatment of CVS and over the first hours after admission to the ICU was accompanied by an increase in blood pressure followed by a decrease in vasopressor requirement. Early MB administration (during operation) in cardiac vasoplegic syndrome may be even more effective. A norepinephrine dose of 0.8 mg/h (0.15 μg*kg-1*min-1) followed by the need to ramp up the dose (≥ 0.1 mg*h-1) may serve as criteria for early MB administration. More prospective and randomized studies are necessary to further investigate the potential of MB administration on CVS after cardiac surgery.

## Data Availability

The datasets used and analyzed for this study are available from the corresponding author upon reasonable request.

## Abbreviations

CI: Cardiac index
CPB: Cardiopulmonary bypass
CVS: Cardiac vasoplegic syndrome
e.g.: Exempli gratia / for example
ICU: Intensive care unit
h: Hour
L/min/m2: Liter per minute per square meter
MAP: Mean arterial pressure
MB: Methylene blue
mmHg: Millimeter quicksilver
mg*h-1: Milligram per hour
mg*kg-1: Milligram per kilogram
PICCO: Pulse contour cardiac output
PAC: Pulmonary arterial catheter / Swan-Ganz-catheter
QM: Quality management
RF: Risk factor
ScvO2: Central venous saturation
SD: Standard deviation
SEM: Standard error of the mean
SVR: Systemic vascular resistance
SVRI: Systemic vascular resistance index
